# Hepatitis E Virus in Farmed Rabbits, Wild Rabbits and Petting Farm Rabbits in the Netherlands

**DOI:** 10.1007/s12560-016-9239-3

**Published:** 2016-05-04

**Authors:** Sara A. Burt, Jorg Veltman, Renate Hakze-van der Honing, Heike Schmitt, Wim H. M. van der Poel

**Affiliations:** 1 Institute for Risk Assessment Sciences - Veterinary Public Health Division, Faculty of Veterinary Medicine, Utrecht University, PO Box 80175, 3508TD Utrecht, The Netherlands; 2 Department of Virology, Central Veterinary Institute, Wageningen University Research, Edelhertweg 15, 8219PH Lelystad, The Netherlands

**Keywords:** Hepatitis E, HEV, Rabbits, Zoonosis

## Abstract

Rabbits have been suggested as a zoonotic source of Hepatitis E virus. Phylogenetic analysis of HEV isolates from farmed, wild and pet rabbits in the Netherlands (23, 0, and 60 % respectively) showed them to be grouped amongst published rabbit HEV sequences and distinct from most human isolates. Dutch rabbits are unlikely to be a zoonotic source.

**Electronic supplementary material:**

The online version of this article (doi:10.1007/s12560-016-9239-3) contains supplementary material, which is available to authorized users.

## Introduction

Hepatitis E virus (HEV) incidence in the Netherlands seems to be on the rise (Koot et al. 2015). About two-thirds of the acute HEV cases in the Netherlands were suggested to be unrelated to travel to endemic countries (Herremans et al. 2007). Domestic pigs have been demonstrated to be a true reservoir (Bouwknegt et al. 2008) and seem to be a main source of infection (Van der Poel et al. 2001), but other species may play a role also (Meng 2010; Rutjes et al. 2014). Recently, rabbit HEV isolates were reported to be phylogenetically closely related to a particular human strain (Caruso et al. 2015; Izopet et al. 2012), suggesting rabbits as another potential source for zoonotic transmission. Since rabbits are widely kept as house pets and are also farmed for food and exist as an extensive wild population throughout the country, the consequences of possible zoonotic transmission of rabbit HEV may be far-reaching. We therefore investigated HEV in rabbits on petting farms, farmed rabbits and wild rabbits in the Netherlands to assess the likelihood of zoonotic transmission.

## Materials and Methods

In 2013 faecal droppings were collected from 35 rabbits at 12 petting farms. Faecal samples and liver samples were collected from 10 farmed rabbits at slaughter. Faecal and liver samples (32 and 30, respectively) were collected from 32 wild rabbits shot by hunters at 11 locations throughout the Netherlands. Samples were stored at −20 °C until analysis. HEV RNA was isolated and Reverse Transcription-PCR for HEV was carried out to amplify a fragment of 70 base pairs using the method of Jothikumar et al. (2006) with adaptations. The RNA primers and probes used targeted the ORF 3 region (starting at bp 27). Primer and probe sequences were as follows: forward primer JVHEVF: 5′ GGTGGTTTCTGGGGTGAC 3′, reverse primer JVHEVR: 5′ AGGGGTTGGTTGGATGAA 3′ and probe JVHEVP: 5′ TGATTCTCAGCCCTTCGC 3′. The presence of a 70-bp-long fragment in the RT-PCR products was confirmed by gel electrophoresis (3 % agarose gel in Tris–borate buffer with EDTA, stained with SybrGold) (Online Resource 1).

RT-PCR amplification products of five samples showing the lowest Ct values (highest RNA concentrations) in the screening RT-PCR (see above) were amplified using a nested RT-PCR format targeting an ORF2 fragment of HEV using a method presented at a meeting of the Dutch Society for Clinical Virology, January 2012, Arnhem, The Netherlands (Online Resource 2). With this PCR, a product of 493 bp was obtained. The sequences of the rabbit isolates were subjected to phylogenetic analysis together with published sequences for human HEV from the Netherlands (healthy blood donors and liver patients) and for rabbit HEV and human HEV from other countries.

## Results

**Fig. 1:**
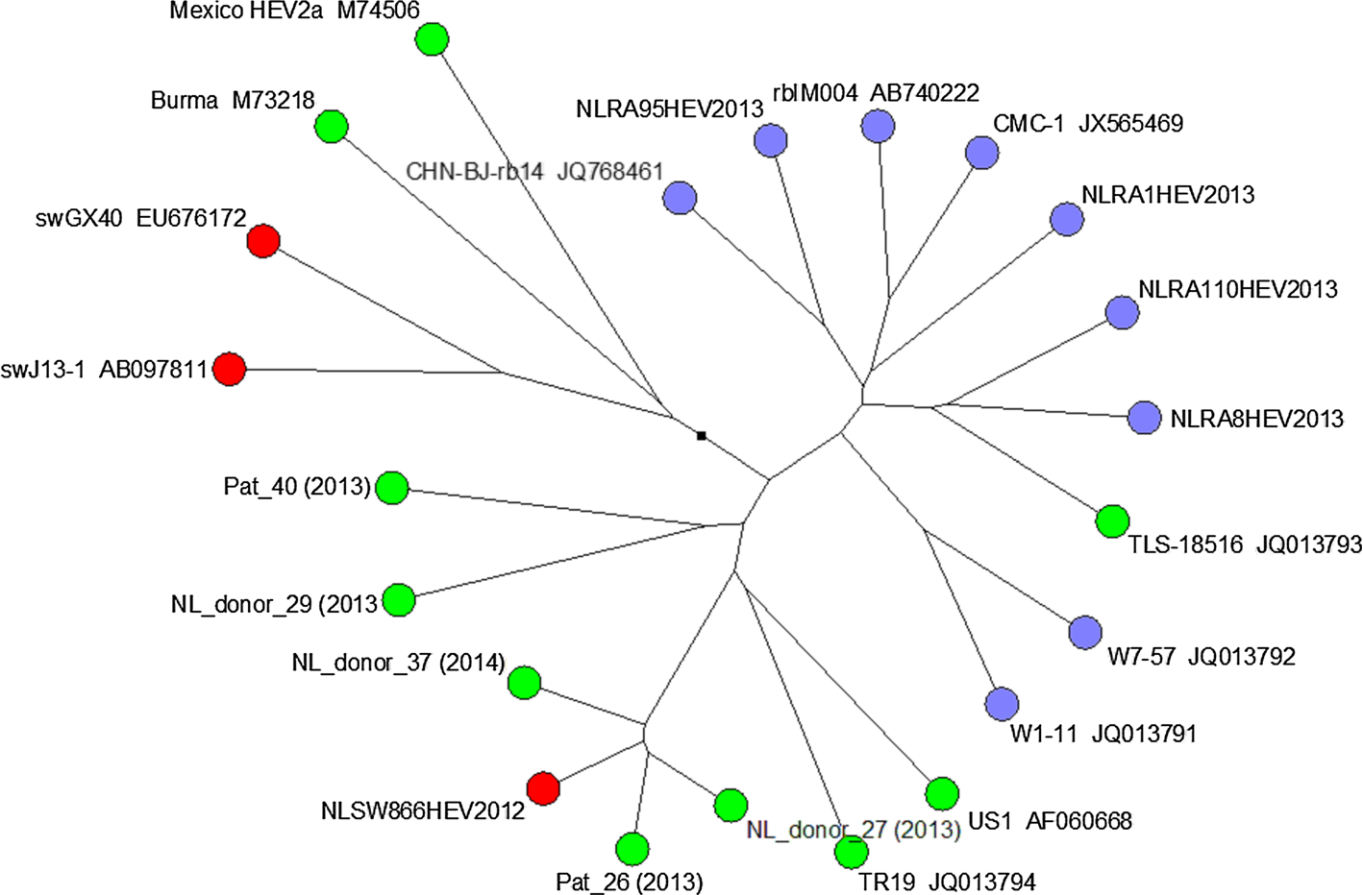
Phylogenetic tree of HEV ORF2 sequences detected in rabbits in the Netherlands (*blue circles*, labelled NLRA) related to HEV sequences of rabbits elsewhere (remaining *blue* circles), humans (*green*) and swine (*red*) reported in the literature

The prevalence of HEV was 8/35 (23 %) in petting farm rabbit droppings, 0/10 (0 %) in farmed rabbits, 5/32 (16 %) in wild rabbit faeces and 18/30 (60 %) in wild rabbit liver samples. Of the five wild rabbits with positive faecal samples, four of these also had positive liver samples. Phylogenetic analysis of PCR amplification products from two pets and two wild rabbits showed that they were located amongst sequences from rabbit isolates from other countries, as shown in Fig. 1. Sequences from Dutch blood donors and other human sequences from other regions do not cluster with the detected Dutch rabbit sequences except one. This concerns an earlier reported sequence of a human liver patient (TLS-18516 (Izopet et al. 2012)).

## Discussion

To the authors’ knowledge, there is only one previous report of HEV in a pet rabbit. A house rabbit that had died suddenly was found to be HEV positive; the sequence similarity was determined to be 82.6–90.1 % with rabbit HEV and 71.4–89.5 % with human HEV (Caruso et al. 2015). HEV prevalence in farmed rabbits is reported to vary from 7 to 15 % as measured by HEV RNA in liver or faeces and 16–55 % as measured by the seroprevalence of HEV-specific antibodies (Cossaboom et al. 2011; Geng et al. 2011; Izopet et al. 2012). The small number of farmed rabbits sampled in the present study (*n* = 10) may have contributed to the lack of HEV-positive faecal samples. In a French study in which faeces from 20 groups of ten farmed rabbits were sampled, 13/20 of these groups were also negative for HEV RNA (Izopet et al. 2012). In the present study, we investigated viral RNA in faeces rather than seropositivity since the faecal–oral route is relevant to possible zoonotic infection. HEV prevalence presented here for wild rabbit livers (18/30 or 60 %) is higher than has been reported in France (47/205 or 23 %) (Izopet et al. 2012). This difference in prevalence may be due to differences in ecology, climate and geography of the two countries. However, differences in the numbers of samples or the methods and primers that were used may also contribute to this difference.

Phylogenetic analysis of PCR amplification products from pets and wild rabbits showed that they were located amongst sequences from rabbit isolates from other countries (Fig. 1). This location is distinct from published human HEV sequences including those from healthy blood donors and liver patients in the Netherlands but it is close to one isolate from a hospital patient in France (Izopet et al. 2012). These results are in agreement with papers that propose a classification of rabbit HEV as a distinct subtype of genotype 3, but this has not yet been adopted (Cossaboom et al. 2012; Smith et al. 2016; Vina-Rodriguez et al. 2015).

In conclusion, phylogenetic analysis of rabbit HEV from pets and wild rabbits showed them to be grouped amongst published rabbit HEV sequences and distinct from most human isolates. Rabbits in the Netherlands are therefore considered unlikely to be a source of zoonotic infection.

## Electronic supplementary material

Below is the link to the electronic supplementary material.

Supplementary material 1 (DOCX 18 kb)

Supplementary material 2 (DOCX 13 kb)
