# Supplementary material for: Hepatitis E Virus in Farmed Rabbits, Wild Rabbits and Petting Farm Rabbits in the Netherlands
Source: Food Environ Virol. 2016 May 4;8(3):227–9. doi: 10.1007/s12560-016-9239-3 (PMC4972841; doi:10.1007/s12560-016-9239-3)
Supplement: Supplementary file 1 — Supplementary material 1 (DOCX 18 kb) [file 12560_2016_9239_MOESM1_ESM.docx]

Online resource 1.

PCR for detection of HEV RNA, adapted from Jothikumar et al. (2006).

The RNA primers and probes used targeted the ORF 3 region (starting at bp 27). Primer and probe sequences were as follows: forward primer JVHEVF: 5’ GGTGGTTTCTGGGGTGAC 3’, reverse primer JVHEVR: 5’ AGGGGTTGGTTGGATGAA 3’, and probe JVHEVP: 5’ TGATTCTCAGCCCTTCGC 3’. The 20μl mastermix consisted of 12μl 2x QuantiTect Probe RT-PCR Master Mix (Qiagen, Venlo, The Netherlands), 0,31μl forward primer (20 pmol/l), 0,31μl reverse primer (20 pmol/l), 0,25μl probe (10 pmol/l), 0,24μl QuantiTect RT enzyme mix (Qiagen) and 6,89μl water. 5μl RNA was added to the Master Mix. The PCR was carried out as follows: reverse transcription at 50°C for 30 minutes, followed by denaturation at 95°C for 15 minutes. The DNA was amplified with 45 cycles at 95°C for 10 seconds, 55°C for 20 seconds en 72°C for 15 seconds. The positive control consisted of a HEV RNA with a known Ct-value and the negative control was 5 µl RNAse free water. The presence of a 70 bp length fragment in the RT-PCR products was confirmed by gel electrophoresis (3% agarose gel in Tris-borate buffer with EDTA, stained with SybrGold).
