# Supplementary material for: Hepatitis E Virus in Farmed Rabbits, Wild Rabbits and Petting Farm Rabbits in the Netherlands
Source: Food Environ Virol. 2016 May 4;8(3):227–9. doi: 10.1007/s12560-016-9239-3 (PMC4972841; doi:10.1007/s12560-016-9239-3)
Supplement: Supplementary file 2 — Supplementary material 2 (DOCX 13 kb) [file 12560_2016_9239_MOESM2_ESM.docx]

Online resource 2.

PCR for sequencing of HEV (Presented at the General meeting, Dutch Society for Clinical Virology, January 2012, Arnhem, The Netherlands.)

RT-PCR amplification products of five samples showing the lowest Ct-values (highest RNA concentrations) in the screening RT-PCR (see above) were amplified using a nested RT-PCR format targeting an ORF2 fragment of HEV. The RT mix contained 2 µl (5X) first strand buffer (Invitrogen, Breda, The Netherlands), 50 mM DTT, 1 mM dNTP mix (TaKaRa, City, Country), 20 U RNasin (Promega, Madison, USA) , 100 U Superscript III reverse transcriptase (Invitrogen, Breda, The Netherlands) and 2,5µM primer HEV222alt: GAR AAi GGR CGi GAi GGR GCi GG. Five µl RNA was added to 5µl RT-mix, thus the RT reaction was performed at a final volume of 10 µl. The mixture was incubated for 10 min at 22o C followed by 60 min at 42o C, heated for 5 min at 95o C and then placed on ice. Forty µl of the PCR1 mixture was added to the RT mixture. The PCR1 mix contained 2,5µM primer HEV224alt: AAY CAR GGi TGG CGY TCi GTi GAR AC, 10 X PCR buffer (Invitrogen, Breda, The Netherlands), 1,5 mM MgCl2, 0.2 mM dNTP and 2.5 U Taq DNA polymerase. The PCR reaction was performed in a final volume of 50 µl. Cycling conditions were denaturation at 95oC for 5 minutes followed by 35 amplification cycles (95oC 30 s, 42oC 30 s and 60oC 45 s). One µl of the first PCR was used in the nested PCR. The PCR2 mix contained 10x PCR buffer (FS Taq polymerase 2161567, Roche), 2,5 U FS Taq polymerase (FS Taq polymerase 2161567, Roche), 0,2mM dNTP and 1µM of each primer HEV-AN89alt: GAG GAG GAA GCT ACC TCY GGY YTi GTi ATG CTY TGY AT and HEV-AN88alt: GGA GAA GGA GTT GGT CGR TCY TGY TCR TGY TGR TT. The PCR reaction was performed in a final volume of 50 µl. Cycling conditions were denaturation at 95o C for 6 min followed by 40 amplification cycles (95°C 30 s, 60°C 20 s, and 72°C 15 s). With this PCR, fragments of 493 nucleotides were obtained. The PCR products were separated in a 1.5% agarose gel and visualized under UV after ethidium bromide staining. Positive RT-PCR products were excised from the gel, purified by using a Gel DNA Recovery kit (Zymo Research, CA, USA), and sequenced in both directions subsequently. For sequencing we used the primers HEV-AN232: GAG GAG GAA GCT ACC TC and HEV-AN233: GGA GAA GGA GTT GGT CG (General meeting, Nederlandse Vereniging voor Klinische Virologie, January 2012, Arnhem). The protocol for the sequence PCR was kindly provided by Harry Vennema (RIVM). Nucleotide sequences were aligned and clustered using Bionumerics version 6.6 (Applied Maths) using the Jukes and Cantor correction for evolutionary rate. Evolutionary trees were drawn using Neighbour-Joining clustering. PCR amplification products from two pet and two wild rabbit samples were sequenced in both directions. Sequences for the nucleotides are available from GenBank under the following accession numbers:

BankIt1912533 Seq1 KX110047

BankIt1912533 Seq2 KX110048

BankIt1912533 Seq3 KX110049

BankIt1912533 Seq4 KX110050
